# Phytochemical Characterization and Mechanistic Insights into the Cytotoxic Effects of *Bougainvillea glabra* Bract Extract in Caco-2 Colorectal Adenocarcinoma Cells

**DOI:** 10.1007/s11130-026-01560-7

**Published:** 2026-08-26

**Authors:** Mariana C. Leal-Alcazar, Frida Bautista-Palestina, María Rocha-Pizaña, Juan L. Monribot-Villanueva, José A. Guerrero-Analco, Luis Mojica, Jonhatan Contreras, Diego A. Luna-Vital

**Affiliations:** 1 Tecnológico de Monterrey, School of Engineering and Science, Ave. Eugenio Garza Sada 2501, Monterrey, Nuevo León 64849 México; 2 Red de Estudios Moleculares Avanzados, Instituto de Ecología, A.C., Carretera Antigua a Coatepec 351, Xalapa, Veracruz 91073 México; 3 Food Technology, Center for Research and Assistance in Technology and Design of the State of Jalisco, A.C. (CIATEJ), Camino Arenero 1227, El Bajío Arenal, Zapopan, Jalisco 45019 México

**Keywords:** *Bougainvillea glabra*, Cytotoxicity, Betalains, Phenolic compounds, Apoptosis, Autophagy, Antioxidant

## Abstract

*Bougainvillea glabra* Choisy (Nyctaginaceae) is a traditional medicinal plant and a source of betalains and phenolic compounds with potential antioxidant and cytotoxic properties that still remains underexplored. This study characterized a *B. glabra* extract (BGE) and evaluated its antioxidant capacity, cytotoxic effects, and modulation of intracellular ROS levels in Caco-2 cells. UPLC-MS identified 21 phenolic compounds, including rutin and quercetin, and two major betacyanins. BGE exhibited ABTS radical scavenging activity (156–188 µM TE), reduced intracellular ROS levels under basal and LPS-stimulated conditions, decreased Caco-2 cell viability, and enhanced the cytotoxic effect of cisplatin. These effects were associated with apoptosis- and autophagy-related markers, including p53 and Bcl-2 expression, and differential regulation of genes involved in caspase activation and NF-kB signaling. Overall, BGE exhibited antioxidant and cytotoxic activities, supporting its potential as a source of bioactive compounds for colorectal cancer research.

**Supplementary Information:**

The online version contains supplementary material available at https://doi.org/10.1007/s11130-026-01560-7.

## Introduction

Colorectal cancer is the third most common cancer worldwide and second leading cause of cancer-related mortality, representing a major public health challenge [1]. Although current therapies, including surgery, chemotherapy, and radiotherapy, have improved patient survival, treatment-associated toxicity remains a significant limitation [2]. Consequently, plant-derived bioactive compounds have attracted attention as complementary therapeutic strategies [3]. Among these, betalains and polyphenols are particularly promising due to their antioxidant, anti-inflammatory, cytotoxic, and chemo sensitizing properties [4, 5].

Betalains are water-soluble pigments classified into red-violet betacyanins and yellow-orange betaxanthins. Beyond their role as natural colorants, betalains exhibit antioxidant, anti-inflammatory, and antiproliferative properties, as they have been associated with apoptotic cell death in different cancer models such as lung, prostate, cervical, breast, and colorectal cancer [6]. In parallel, polyphenols comprise a diverse group of plant secondary metabolites widely recognized for their antioxidant, anti-inflammatory, and anti-cancer properties [7]. Particularly, rutin and quercetin have been recognized for their modulation of key pathways in colorectal cancer, as well as to exert antioxidant and anti-inflammatory activity [4]. The coexistence of betalains and polyphenols in plant matrices suggests potential cooperative interactions, broadening therapeutic potential beyond what individual compounds achieve.

*Bougainvillea glabra*, known in Spanish as Santa Rita, Flor de papel, and papelillo, is a Latin American ornamental plant traditionally used to treat respiratory and inflammatory conditions [8]. Its bracts contain flavonoids, other phenolic compounds, and betalains (bougainvilleins), and recent studies have demonstrated the cytotoxic potential of *B. glabra* extracts against several cancer cell lines, including cervical, prostate, breast, colon, and lung cancers [8–11]. However, to date, their effects have not been evaluated in Caco-2 cells, a well-established and widely used in vitro model for evaluating cytotoxic and mechanistic responses to bioactive compounds [12]. Given the contribution of oxidative stress and chronic inflammation to colorectal carcinogenesis through ROS production and inflammatory mediators [13, 14], investigating both the antioxidant and cytotoxic properties of *B. glabra* may provide further insight into their therapeutic potential.

This study aimed to characterize the phytochemical profile of a betalain-rich extract (BGE) from *B. glabra* bracts and evaluate its antioxidant activity, cytotoxic effects, and modulation of intracellular ROS levels in Caco-2 cells, including its activity in combination with cisplatin.

## Materials and Methods

The material and methods section are provided as Supplementary online resource.

## Results

### Phytochemical Characterization of *Bougainvillea glabra* Extract (BGE)

The UV-Vis spectrum of BGE showed absorption maxima regions 280 and 530 nm (Fig. S1). Spectrophotometric analysis estimated a total phenolic content (TPC) of 2 mg/g DW, whereas UPLC-MS identified 21 phenolic compounds with a combined concentration of 16.78 mg/g DW (Table 1; analytical conditions provided in Table S1). Flavonoids were the predominant class, including rutin, quercetin-3-D-galactoside, and quercetin-3-glucoside as the major compounds, while phenylalanine was also detected at high levels. Untargeted profiling confirmed the presence of rutin, quercetin glucosides, and several isorhamnetin derivatives (Table S2). Ethanolic extraction yielded 0.118 mg of total betalain content (TBC) /mg of dry weight (14.46%), and two betacyanins were detected by UPLC-MS (Fig. S2).

**Table 1:** Identification and quantification of phenolic compounds in *Bougainvillea glabra* extract (BGE) by UPLC-MS using authentic reference standards

Type	Name	Concentration (µg/g of dried extract)
Precursor	Phenylalanine	1352.52 ± 84.33
Phenolic acids	4-Coumaric acid	475.21 *±* 20.96
	Caffeic acid	131.99 *±* 12.31
	Salicylic acid	124.33 *±* 1.11
	Sinapic acid	53.63 *±* 2.41
	Vanillic acid	52.71 *±* 1.41
	4-Hydroxybenzoic acid	32.23 *±* 0.39
	Ferulic acid	29.56 *±* 1.35
	Protocatechuic acid	21.26 *±* 0.23
	Gentisic acid	19.78 *±* 0.21
	Gallic acid	16.45 *±* 0.32
	*t*-Cinnamic acid	5.40 *±* 0.35
Hydroxycoumarin Phenolic aldehyde	Umbelliferone Vanillin	2.40 *±* 0.12 6.47 *±* 0.77
Flavonoids	Rutin	6117.59 *±* 442.06
	Quercetin-3-D-galactoside	5778.66 *±* 583.70
	Quercetin-3-glucoside	2459.95 *±* 233.91
	Kaempferol-3-O-glucoside	51.22 *±* 1.70
	Quercetin	38.23 *±* 0.79
	Kaempferol	5.33 *±* 0.17
	(-)-Epicatechin	0.52 *±* 0.03*

*Below the limit of quantification. ± Represents the range of variability around the mean.

### In vitro Antioxidant Capacity and ROS Modulation of BGE

**Fig. 1:**
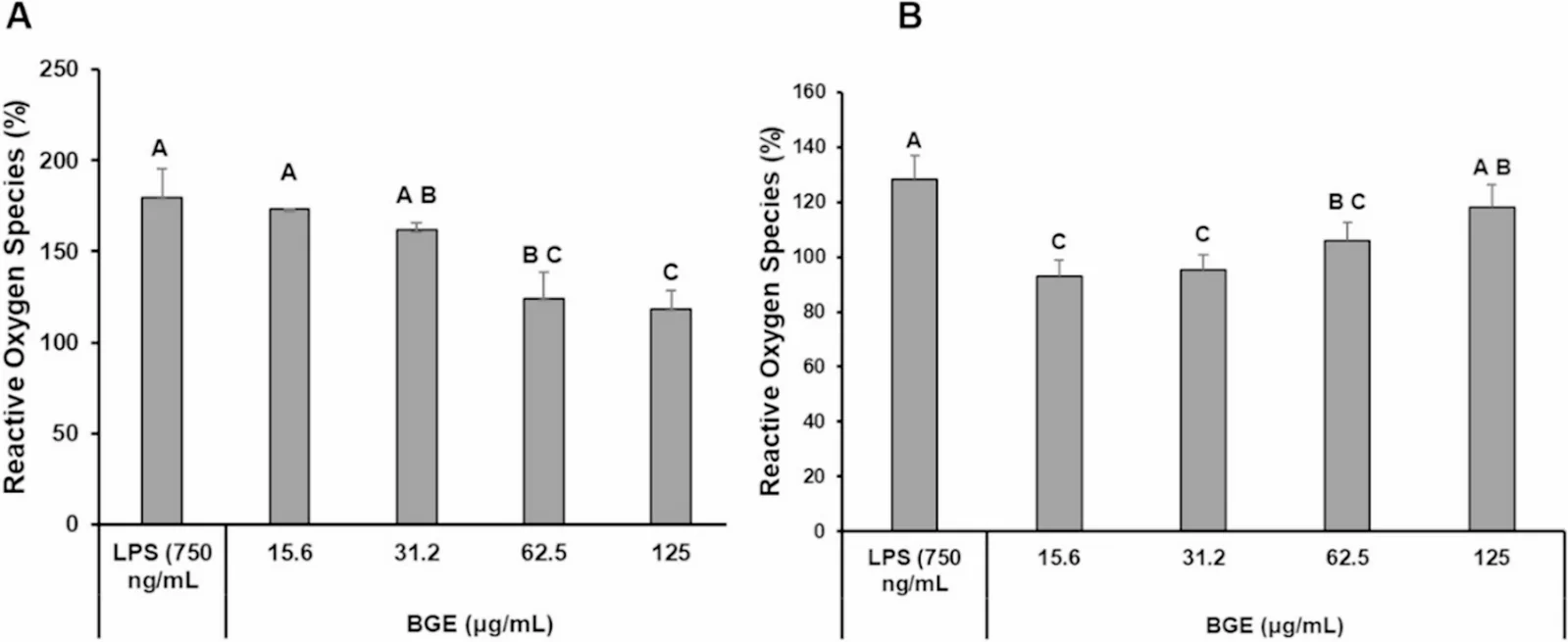
Effect of BGE on intracellular reactive oxygen species (ROS) levels in LPS-stimulated macrophages. (**A**) Pre-treatment with BGE before LPS stimulation. (**B**)Post-treatment with BGE after LPS stimulation. ROS levels were normalized to LPS-treated control. Different letters indicate statistically significant differences according to Tukey’s *post hoc* test (*p* < 0.05)

BGE exhibited strong ABTS radical scavenging activity at all tested concentrations (165.74 to 188.9 µM TE), with the highest activity observed at 0.125 and 0.250 mg/mL (Table 2). Regarding intracellular ROS modulation, two experimental approaches were evaluated. In cells pre-treated with BGE prior to LPS stimulation (Fig. 1A), higher concentrations significantly attenuated ROS levels, with the most pronounced effect at 125 µg/mL, which significantly decreased ROS compared to the LPS-stimulated control. Lower concentrations showed either no significant effect (15.6 µg/mL) or intermediate responses without consistent statistical separation (31.2 and 62.5 µg/mL). In LPS-stimulated macrophages subsequently treated with BGE (Fig. 1B), BGE significantly reduced ROS levels at low concentrations (15.6 and 31.2 µg/mL) compared to LPS-stimulated control cells. However, this effect was not maintained at higher concentrations, as 62.5 and 125 µg/mL did not differ significantly from the LPS control, indicating a non-linear concentration response.

**Table 2:** Trolox equivalent (TE) and percentage inhibition of ABTS radical scavenging test of *Bougainvillea glabra extract* (BGE) at different concentrations

Sample Concentration [mg/mL]	Inhibition (%)	Trolox Equivalent (TE) [µM]
1.0	91.9 ± 0.0034 ^B^	165.74^B^
0.50	88.4 ± 0.01245^B^	156.59^B^
0.25	100.0 ± 0.00226^A^	188.90^A^
0.125	98.8 ± 0.00226^A^	183.11^A^

± Represents the range of variability around the mean. Different letters indicate statistically significant differences according to Tukey’s *post hoc* test (*p* < 0.05). Trolox calibration curve available in Fig. S3

## Cytotoxic Activity of BGE Against Caco-2 Cells and Combined Effect with Cisplatin

**Fig. 2:**
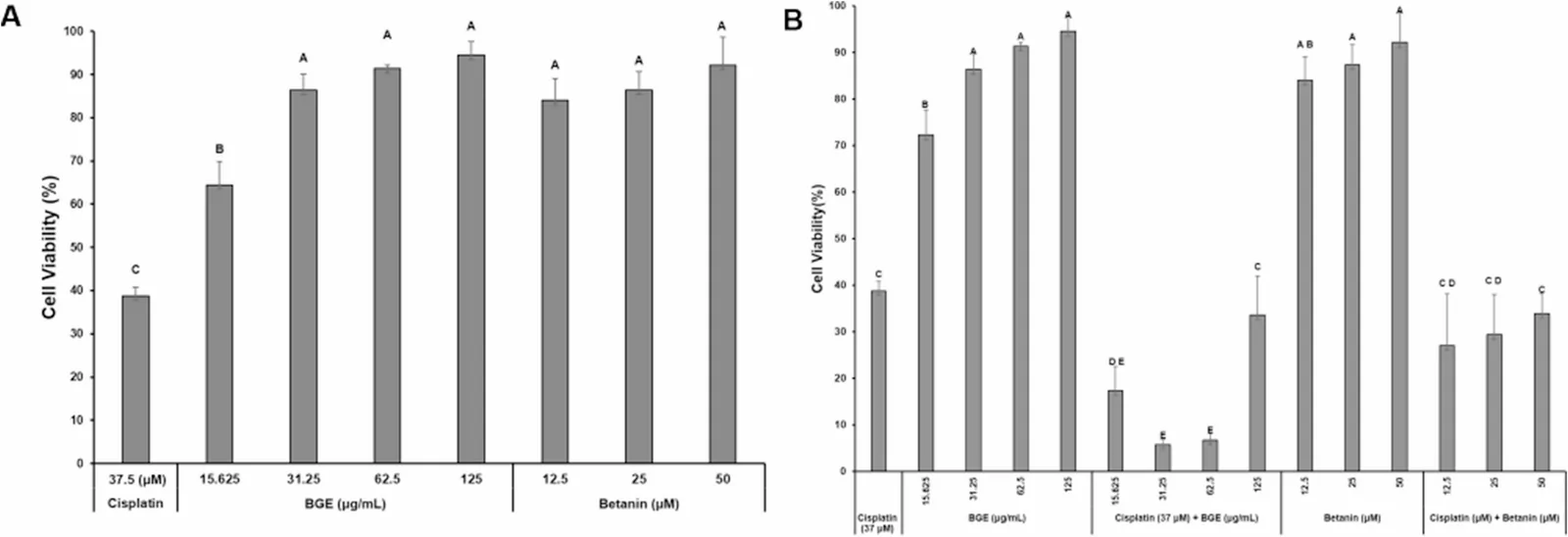
Effects of BGE, betanin, and cisplatin on Caco-2 cell viability and combined treatment (**A**) Cell viability after treatment with BGE, betanin, and cisplatin (37.5 µM) for 48 h. (**B**) Combined effects of cisplatin with BGE or betanin on Caco-2 cell viability. (37.5 µM final concentration) for 48 h. Data are presented as mean ± SD (*n* = 3). Different letters indicate statistically significant differences according to Tukey’s *post hoc* test (*p* < 0.05)

BGE showed no significant cytotoxic effect toward HDFa cells, maintaining viability above 80% at all tested concentrations, whereas cisplatin reduced HDFa viability by approximately 50% (Fig. S4). In Caco-2 cells, both BGE and purified betanin decreased cell viability (Fig. 2A). The greatest effect was observed with 15.625 µg/ml BGE, while higher concentrations produced no additional reduction in viability. Co-treatment with cisplatin further reduced Caco-2 cell viability compared to cisplatin alone (Fig. 2B), particularly at lower and intermediate BGE concentrations. The strongest combined effect was observed with 37 µM cisplatin combined with 31.25 µg/mL BGE, whereas the highest concentration tested (125 µg/ml) showed a comparatively weaker effect. Overall, most combinations yielded measured-to-expected ratios above 1.0, suggesting potential cooperative interactions (Table S3).

## Changes in Autophagy and Apoptosis-associated Markers following BGE Treatment

**Fig. 3:**
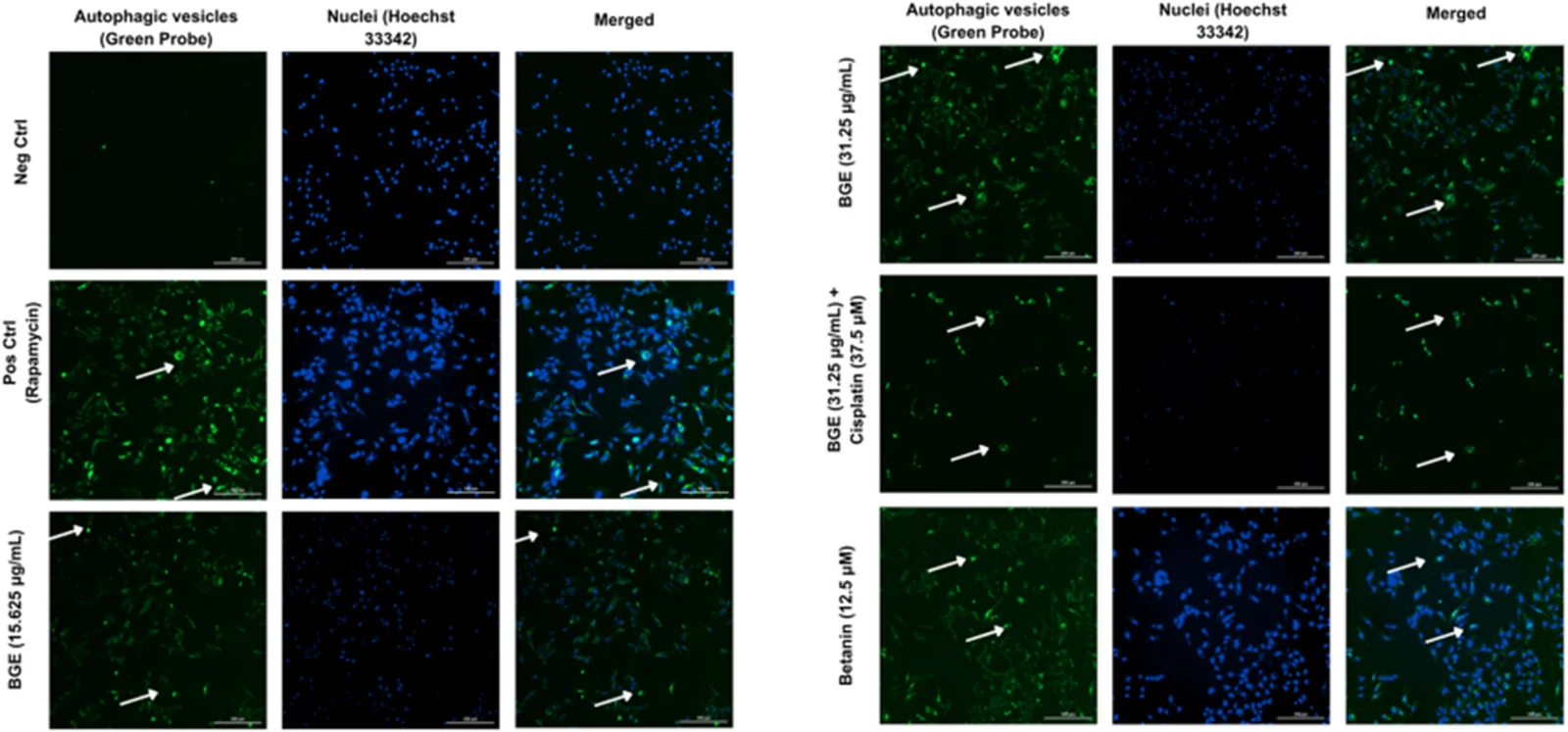
Formation of autophagic vesicle in Caco-2 cells treated with BGE, betanin, cisplatin, and their combinations. Representative fluorescence images of autophagic vesicles (green) and Hoechst-stained nuclei (blue). White arrows indicate autophagic vesicles accumulation. Fluorescence was normalized to Hoechst-positive nuclei. Scale bar = 300 μm

BGE increased autophagy-associated fluorescence in Caco-2 cells, particularly at 31.25 and 62.5 µg/ml (Fig. S5). Increased fluorescence was also observed in rapamycin- and betanin-treated cells, whereas co-treatment with cisplatin showed elevated fluorescence despite the reduced cell density under this condition (Fig. 3 and S5).

**Fig. 4:**
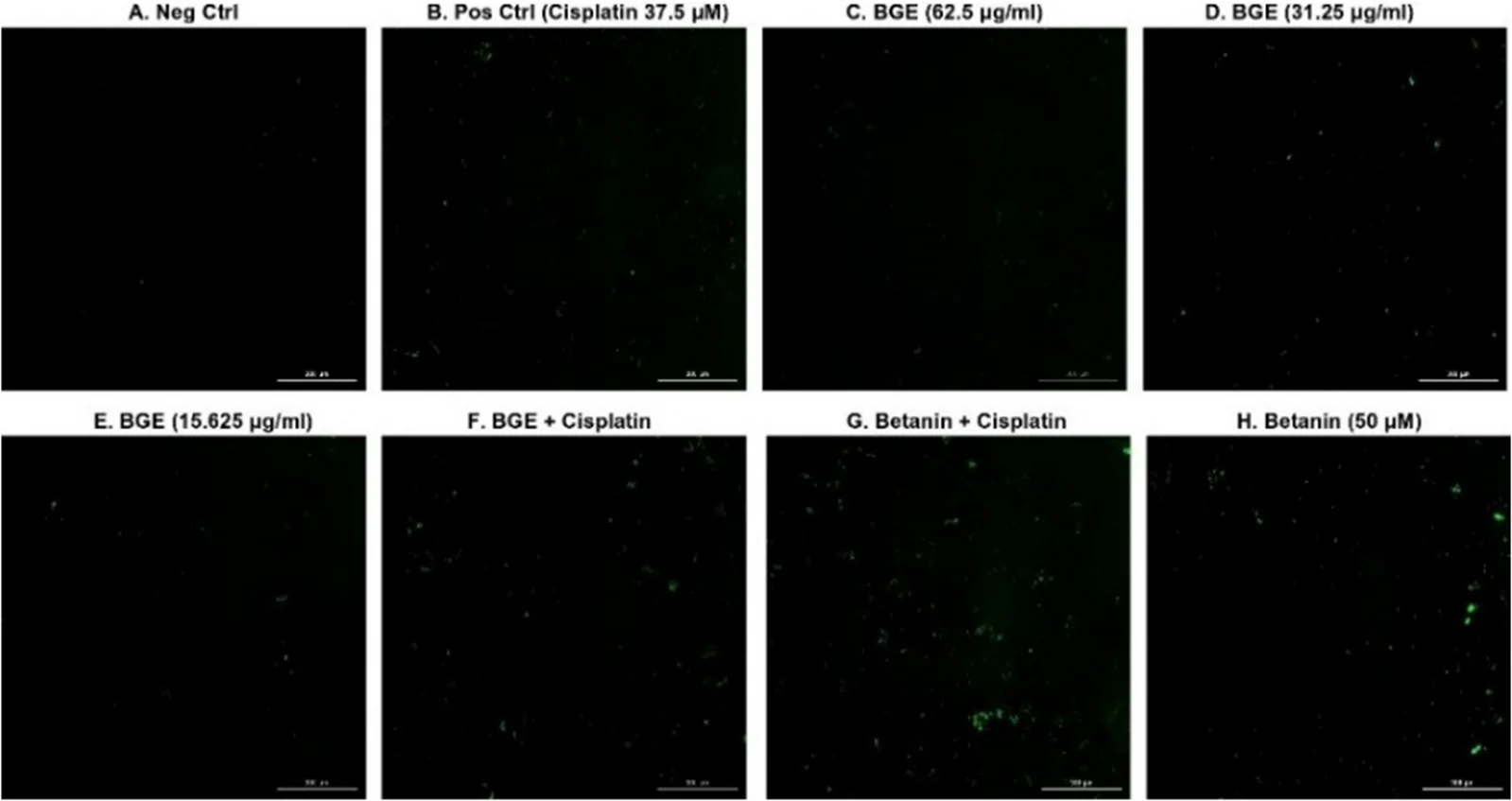
Apoptosis-associated fluorescence staining in Caco-2 cells treated with BGE, betanin, cisplatin, and their combinations. Representative fluorescence microscopy images showing apoptotic-associated green fluorescence. Scale bar = 300 μm

Annexin V-FITC staining showed a trend toward higher levels at 48 h of BGE treatment, although no significant differences were observed among BGE concentrations (Fig. 4). Cisplatin- and betanin-treated cells also exhibited increased Annexin V staining, indicating a shift toward early apoptotic populations (Fig. S6). Betanin showed the highest fold change among treatments; however, this was not significantly different from BGE.

At the protein level, all treatments increased p53 expression in the representative plot, with the highest levels observed in the cisplatin combination groups, accompanied by alterations in the expression patterns of apoptosis-related proteins, including variable levels of Bcl-2 (Fig. S7). TaqMan Apoptosis Array analysis identified differential expression of CASP6, IFT57 and REL (upregulated), whereas CFLAR, IKBKG, and TNFRSF21 were downregulated (Table 3).

**Table 3:** Relative expression changes of genes associated with apoptosis, autophagy, and oxidative stress following BGE treatment in Caco-2 cells

Gene	Fold Change	Significance	Function
CASP6	4.77	Up	Effector protease that cleaves structural and nuclear proteins
CFLAR	-1.19	Down	Apoptosis inhibitor by binding to FADD, inhibiting FADD-caspase 8 interaction
IFT57	1.87	Up	Protein for intraflagellar transport (cilia and flagella) and vesicle trafficking
IKBKG	-3.51	Down	NF-kB signaling pathway regulator
REL	1.17	Up	NF-kB transcription factor
TNFRSF21	-1.31	Down	Death receptor for extrinsic apoptosis activation

## Discussion

While the strong absorption at 280 nm suggested the presence of phenolic compounds, TPC analysis was considerably lower than the UPLC-MS estimated quantification. This discrepancy likely reflects methodological differences, as the Folin-Ciocalteu assay is a non-specific redox-based method that estimates global reducing capacity [15]. Differences in extraction and ionization efficiency, matrix effects, and metabolite coverage may further contribute to the higher values obtained by UPLC-MS, providing complementary but not directly comparable estimates of phenolic content.

Flavonoids were the predominant phenolic class in BGE, consistent with previous reports for *Bougainvillea* species [11, 16, 17] while phenylalanine, detected at notably high levels, further supports its central role as a precursor of polyphenols, flavonoids, and betalains. The rutin content in BGE was considerably higher than previously reported for methanolic *B. glabra* bract extracts and varied from values described for other *Bougainvillea* species [18, 19]. Likewise, the 4-coumaric acid concentration greatly exceeded earlier reports [18]. These differences likely reflect variations in extraction methodology, plant material, and environmental conditions, highlighting the strong influence of extraction procedures on the phytochemical profile of *B. glabra.* In addition, flavonols derived from quercetin, isorhamnetin, and their derivatives detected in BGE (Table S2) may further contribute to antioxidant and anti-inflammatory activities [4, 20].

The TBC yield was lower than previously reported for ultrasound-assisted extraction [21], highlighting the strong influence of extraction solvent on betalain recovery. Although methanol has been reported to improve extraction, ethanol remains the preferred solvent for food and pharmaceutical applications [9]. In this study, only two betacyanins were identified (Fig. S2), consistent with previous reports under similar extraction conditions [16, 22]. These findings indicate that both methodological and biological factors influence the betalain profile of *B. glabra.*

The strong ABTS radical scavenging activity is consistent with previous reports for *B. glabra* and *B. spectabilis* extracts [9, 21, 23]. A non-linear response pattern, with higher scavenging activity at lower concentrations, was observed. This behavior may reflect methodological and matrix-related factors commonly associated with colorimetric antioxidant assays, including optical reference, assay saturation, and complexity of phytochemical mixtures [24]. Although measurements were performed within the assay working range (Fig. S3) and background absorbance correction, the inverse concentration-response should be interpreted cautiously, as phytochemical interactions and the limitations of chemical antioxidant assays may not fully represent in vivo antioxidant behavior.

BGE modulated intracellular ROS levels in LPS-stimulated macrophages in a concentration and treatment-dependent manner. In pre-treated cells, BGE exerted a partial protective effect, with the greatest reduction in ROS observed at 125 µg/mL (Fig. 1A), whereas lower concentrations showed limited or intermediate effects. In contrast, post-treatment reduced ROS predominantly at 15.6 and 31.2 µg/mL, while higher concentrations were not significantly different from the LPS-stimulated control. These findings are consistent with the antioxidant properties of betalain-rich extracts, which can directly scavenge reactive oxygen species and enhance endogenous antioxidant defenses through the activation of antioxidant enzymes and NRF2 signaling [13]. Together with the high betacyanin content of BGE [19], these mechanisms may explain its protective effects under inflammatory conditions.

The lack of significant cytotoxic effects on normal HDFa cells, suggests potentially selective cytotoxic activity toward cancer cells. This finding is consistent with previous reports showing minimal toxicity of *Bougainvillea* extracts in normal cells and greater cytotoxicity in cancer cell lines [25]. Such selectivity has been attributed to the altered membrane properties and tumor microenvironment of cancer cells, which may facilitate the uptake of phytochemicals, including betalains [26]. A limitation of this study was the absence of a clear concentration-dependent response. Lower concentrations occasionally produced effects comparable to or greater than those at higher concentrations, possibly reflecting the complex composition of the extract and interactions among its constituents.

The greater cytotoxic efficacy of crude BGE compared to purified betanin suggests potential additive or synergistic interactions among betalains and flavonoids. However, the measured-to-expected ratio approach used in this study provides only preliminary evidence of potential cooperative interaction and does not replace standardized pharmacological synergy analyses such as Bliss independence, Loewe additivity, or Chou–Talalay modeling.

The cytotoxic activity of BGE is consistent with previous studies reporting antiproliferative effects of *Bougainville*a and other betalain-rich extracts in different cancer cell lines^,^ [12, 18]. Beyond betalains, flavonoids such as rutin and quercetin derivatives may further contribute to the cytotoxic activity of BGE through the modulation of signaling pathways involved in cell proliferation, survival, and apoptosis [17].

The potential combined inhibitory effect between BGE and cisplatin is supported by previous studies demonstrating synergistic effects of betalain-rich extracts with conventional chemotherapeutics through enhanced apoptosis, oxidative stress modulation, and inhibition of survival pathways [27–30]. Although the interaction between BGE and cisplatin requires confirmation using standardized synergy analyses, the combined treatment reduced Caco-2 cell viability more effectively than either treatment alone.

The increased autophagy-associated fluorescence observed in BGE-treated Caco-2 cells is consistent with the previous reports describing the ability of betalains to modulate autophagy-related pathways. The greater fluorescence induced by purified betanin compared to crude BGE suggests that individual betalains may contribute more directly to autophagy induction [22]. Previous studies have shown that indicaxanthin promotes autophagolysosome formation and increases LC3-II and Beclin-1 expression, possibly through epigenetic regulation and interaction with Bcl-2 [31]. Similarly, betanin and isobetanin have been associated with autophagy-related cell death under oxidative stress [32]. These findings support the potential role of betalains as modulators of redox-dependent autophagy, although additional studies using autophagy-specific markers and flux assays are required to confirm the mechanisms involved [33].

On the other hand, BGE-treated cells exhibited increased Annexin V-FITC staining after 48 h, suggesting progressive apoptotic changes over time. However, differences among concentrations were not consistently significant, indicating that early apoptosis was modest under the experimental conditions. Because PI staining could not be evaluated and apoptosis-associated fluorescence was assessed semi-quantitatively, these findings should be considered exploratory and require confirmation using complementary methods.

Previous studies have indicated that betanin and betalain-rich extracts have been associated with apoptotic responses through both intrinsic and extrinsic pathways, modulating Bcl-2, FAS receptor, and caspase-8 expression [32]. The observed changes in apoptosis and autophagy-associated markers suggest an interplay between these processes in colorectal cancer. Preliminary changes in p53 protein expression are consistent with the activation of pro-apoptotic signaling [34] while reduced Bcl-2 expression may favor both apoptosis and autophagy through Beclin-1 regulation, but should be interpreted cautiously as they were obtained from a single independent experiment. Gene expression analysis further supported the activation of apoptosis- and autophagy-related pathways. Upregulation of CASP6 extends the apoptotic potential of BGE beyond caspase-3, possibly amplified by autophagic activity and increased cellular sensitization, while CFLAR downregulation suggests enhanced apoptotic signaling, by potentially relieving inhibition of caspase-8 and increasing LC3 accumulation and autophagosome formation [31]. IFT57 upregulation further supports the autophagy-apoptosis crosstalk suggested, consistent with recent evidence showing that increased IFT protein expression is associated with autophagy vesicle formation and apoptotic regulation [35]. In addition, modulation of NF-κB-related genes (IKBKG and REL) and TNFRSF21 indicates coordinated regulation of survival, inflammatory, and receptor-mediated signaling pathways. Together, these findings suggest that betalains and phenolic compounds may modulate interconnected apoptotic, autophagic, and NF-κB-dependent mechanisms, although additional mechanistic studies are required to validate these pathways.

## Conclusion

*Bougainvillea glabra* is a source of betalains and phenolic compounds exhibiting antioxidant and cytotoxic activities in colorectal cancer-relevant models. BGE reduced Caco-2 cell viability, modulated intracellular ROS levels, and was associated with changes in key molecular markers related to apoptosis and autophagy, including p53, Bcl-2, and genes involved in caspase activation and NF-κB signaling. Overall, these findings support the potential of *B. glabra* extracts to modulate ROS levels and cell death-related pathways in colorectal cancer models.

## Supplementary Information

Below is the link to the electronic supplementary material.

Supplementary Material 1

## Data Availability

No datasets were generated or analysed during the current study.
