# Supplementary material for: Phytochemical Characterization and Mechanistic Insights into the Cytotoxic Effects of Bougainvillea glabra Bract Extract in Caco-2 Colorectal Adenocarcinoma Cells
Source: Plant Foods Hum Nutr. 2026 Aug 26;81(3):115. doi: 10.1007/s11130-026-01560-7 (PMC13518437; doi:10.1007/s11130-026-01560-7)
Supplement: Supplementary file 1 [file 11130_2026_1560_MOESM1_ESM.docx]

**Phytochemical characterization and mechanistic insights into the cytotoxic effects of *Bougainvillea glabra* bract extract in Caco-2 colorectal adenocarcinoma cells**

**Authors and affiliations**

Mariana C. Leal-Alcazar^a^, Frida Bautista-Palestina^a^, María Rocha-Pizaña^a^, Juan L. Monribot-Villanueva^b^, José A. Guerrero-Analco^b^, Luis Mojica^c^, Jonhatan Contreras^c^, Diego A. Luna-Vital^a^*

^a^Tecnológico de Monterrey, School of Engineering and Science, México. Ave. Eugenio Garza Sada 2501, Monterrey, N.L., México, 64849.

^b^Red de Estudios Moleculares Avanzados, Instituto de Ecología, A. C., Carretera Antigua a Coatepec 351, Xalapa, Veracruz, México, 91073.

^c^Food Technology, Center for Research and Assistance in Technology and Design of the State of Jalisco, A.C. (CIATEJ), Camino Arenero 1227, El Bajío Arenal, Zapopan, Jalisco, México, 45019.

**Corresponding author:** dieluna@tec.mx (Luna-Vital, Diego A.)

**Supplementary Information**

Additional data and methods supporting this work.

**Materials and Methods**

**Chemicals and Reagents**

DPPH(2,2- Diphenyl-1-picrylhydrazyl), ABTS ((2,2′-Azino-bis(3-ethylbenzothiazoline-6-sulfonic acid), Trolox, DNS (3,5-Dinitrosalicylic acid), Folin–Ciocalteu’s reagent, gallic acid, DMEM (low glucose, phenol red-free, and DMEM/F-12), and betanin (dextrin-diluted) were obtained from Sigma Chemical Co. (St. Louis, MO, USA). Methanol and sodium carbonate (Na₂CO₃) were purchased from J.T. Baker (Phillipsburg, NJ, USA). The Autophagy Assay Kit and Annexin V-FITC Apoptosis Detection Kit were from Abcam (Cambridge, UK). Antibodies against Bcl-2 and p53 were obtained from Santa Cruz Biotechnology (Dallas, TX, USA), β-actin (JF53-10, HRP) from Invitrogen (Waltham, MA, USA), and secondary anti-mouse IgG HRP from Abcam. The TaqMan® Human Apoptosis Array was purchased from Thermo Fisher Scientific (Waltham, MA, USA).

**Plant Material**

Dried bougainvillea *(Bougainvillea glabra)* flowers were purchased from the greenhouse “Hortisen” in Xalpatlaco, Atlixco, Puebla, México. Before extraction, the bracts were separated from the stems and oven-dried at 40°C for 60 minutes. Bracts were ground with a commercial coffee grinder and sieved to obtain a powder, which was stored at -20°C until further use.

**Ultrasound-assisted Pigment Extraction**

Bougainvillea extract (BGE) was obtained using an ethanol–water (1:1 v/v) solvent mixture at a 1:10 (w/v) solid-to-solvent ratio. The mixture was processed with a probe sonicator (Branson Digital Sonifier®) with a pulse of 20 sec on/5 sec off for 15 min at 60% amplitude. The mixture was centrifuged at 3000 rpm for 10 minutes. The supernatant was concentrated with a rotary evaporator (HAHNSHIN Scientific CO) for 3 hours at 60°C and stored at -80°C until further use.

**Total Betalain content**

The total betalain content (TBC) was determined through Nilsson’s spectrophotometric method using a microplate reader (BioTek Cytation 5, Agilent, USA). Absorbance values at 480 and 530 nm were used to calculate TBC as:

TBC (mg/mL) = (A x DF x MW x 1000)/(0.38 x ɛ)	(1)

where A= absorbance, DF= dilution factor, MW= molecular weight, ɛ= molar extinction coefficient (Bc= 6.5 x 104 L mol ^–1^ cm ^–1^ , Bx= 4.8 x 104 L mol ^–1^ cm ^–1^). Total phenolic content (TPC) was measured by the Folin-Ciocalteu method proposed by Singleton & Rossi [1] with absorbance recorded at 760 nm. Results were expressed as gallic acid equivalents (GA eq).

**Identification and quantification of phytochemicals by Ultra Performance Liquid Chromatography (UPLC) – Mass Spectrometry (MS)**

Compounds included in the targeted phytochemical analysis were identified and quantified using authentic reference standards through comparison of retention times, mass spectral data, and calibration parameters obtained by UPLC-MS analysis. Phenolic compounds in BGE were identified and quantified following established protocols [2]. BGE was analyzed by UPLC coupled to a triple Quadrupole mass spectrometer (Agilent 1290 Infinity/6460 QqQ, Agilent Technologies, St. Clara, CA, USA). Chromatographic separation was performed on a C18 Agilent Eclipse Plus column (2.1 × 50 mm, 1.8 μm) at 40°C with a flow rate of 0.3 mL/min and a 2 μL injection volume. The mobile phase consisted of water/formic acid 0.1% (A) and acetonitrile/formic acid 0.1% (B) with a gradient from 99% A to 99% B over 40 minutes. Mass spectrometry parameters were as follows: gas temperature 300°C, gas flow of 5 L/min, nebulizer pressure of 45 psi, sheath gas at 250°C with 11 L/min flow, capillary voltage of 3500 V, and nozzle voltage of 500 V.

In contrast, compound annotation in the non-targeted profiling analysis was considered tentative when identification was based on accurate mass measurements, fragmentation patterns, and database/literature comparison without confirmation using authentic reference standards. Non-targeted chemical profiling was conducted using UPLC coupled to a Quadrupole time of flight mass spectrometry (QTOF, G2Si, Waters ™, Milford, MA, USA) with separation on an Acquity BEH column (1.7 μm, 2.1 × 50 mm) at 40°C, with a mobile phase gradient from 1% to 99% acetonitrile over 20 minutes, followed by isocratic and re-equilibration steps. The flow rate was 0.3 mL/min with a 5 μL injection. Mass spectrometry was performed in positive electrospray ionization mode with capillary, sampling cone, and source offset voltages set at 3000, 40, and 80 V, respectively. Mass accuracy was monitored throughout acquisition using internal reference, and only features with a measured mass error ≤ 5 ppm were retained for annotation. Data were processed with MassLynx software (v4.1, Waters ™, Milford, MA, USA).

**ABTS radical scavenging**

Antioxidant capacity was evaluated using the ABTS radical scavenging assay with Trolox as a standard. Samples (0.125 to 1 mg/mL) were incubated with ABTS (7 mM) for 5 minutes, and the absorbance was read at 734 nm. Radical scavenging was expressed as

% inhibition = ((*Abs* _control_ – *Abs* _sample_)/(Abs _control_)) x 100	(2)

Extract blanks were included to correct for background absorbance attributable to intrinsic pigment color. Measurements were performed within the linear range of the ABTS assay.

**Intracellular reactive oxygen species (ROS)**

Intracellular reactive oxygen species (ROS) were measured in RAW 264.7 cells (5x10^4^ cells, 48h, 37°C) using the DCFH-DA probe. Two treatment strategies were evaluated: (i) preventive, in which cells were treated with BGE (15.62-125 μg/mL) for 24h prior LPS stimulation (750 ng/mL) for an additional 24h, and (ii) corrective/therapeutic approach, in which cells were first exposed to LPS stimulation for 24h, followed by BGE treatment for 24 h. Experiment controls included untreated cells and LPS-only positive controls. After treatments, cells were incubated with a DCFH-DA (5 μM) for 25 min at 37°C in the dark, and fluorescence was measured at Ex ≈ 485 nm and Em ≈ 520 nm. ROS levels were normalized relative to the corresponding LPS-stimulated control group for each experimental condition. Intracellular ROS was calculated as:

% of control: % ROS = (*FI*_sample_ / *FI*_control_) × 100	(3)

**Cell culture**

Caco-2 colorectal adenocarcinoma (HTB-37), HDFa human dermal fibroblast (PCS-201-012), A549 human lung carcinoma (CCL-185), HepG2 hepatocellular carcinoma (HB-8065), MCF7 human breast carcinoma (HTB-22) and RAW 264.7 macrophage cell (TIB-71) lines were obtained from the American Type Culture Collection (ATCC, Rockville, USA) and grown on DMEM (low glucose) + 10% FBS and 1% Antibiotic/Antimycotic, and DMEM F12 + 10% FBS and 1% Antibiotic/Antimycotic (RAW 264.7) at 37°C, and 5% CO_2_.

**Cell Viability assay**

Caco-2, A549, HepG2, MCF7, and HDFa (non-cancerous cells) were seeded at 2.5 x 10^4^ on 96-well plates and treated with BGE (15.625 to 125 μg/mL), a purified betanin standard (12.5 to 200 μM), cisplatin (18.75 to 75 μM, positive control), or vehicle (negative control). HDFa cell line was included to assess the selectivity and potential cytotoxicity of experimental samples. Cells were incubated for 48 h at 37°C with 5% CO_2_ in DMEM. After that, cell viability was assessed using the CellTiter-Glo assay [3]. The absorbance was measured at 490 nm, and the cell viability was expressed as the percentage of viability against the total viable cells in the negative control.

**Biological interaction between *Bougainvillea extract* (BGE) and chemotherapeutics**

Combinations were prepared using the IC_50_ of cisplatin (37.5 μM) with BGE (15.625 to 125 μg/mL), or betanin (6.25 to 50 μM). Cells were incubated for 48 h at 37°C with 5% CO_2_, and cell viability and growth inhibition were assessed using the CellTiter-Glo assay. Synergy was assessed by the ratio of the measured to the expected cytotoxicity, where the expected cytotoxicity is the sum of individual effects [4]. Ratio > 1.0 indicates synergy, < 1.0 indicates antagonism, and Ratio =1.0 indicates additivity [4]. This approach was used as a preliminary exploratory analysis and does not represent a standardized pharmacological synergy model.

**Autophagy and apoptosis assay**

Autophagic vacuole formation was assessed in Caco-2 cells (2 × 10^5^) using the Autophagy Assay Kit (Abcam, Cambridge, UK). Cells were treated with BGE (15.623, 31.25, and 62.5 μg/mL), rapamycin (500 nM), cisplatin (18.75 μM), purified betanin (12.5 and 25 μM), combinations with cisplatin, or DMEM control for 48 h with treatments and for 24 h with rapamycin. Fluorescence intensity was quantified using Fiji/ImageJ software. Integrated fluorescence density of the autophagy probe was normalized to the number of Hoechst-positive nuclei in the corresponding field. Quantification was performed using two representative fields per condition and expressed as fold change relative to untreated control cells.

Apoptosis was quantified using the Annexin V-FITC Apoptosis Staining / Detection Kit (Abcam, Cambridge, UK) with adaptations for adherent Caco-2 cells and 96-well plate analysis, including staining directly in the wells, adjusting reagent volumes, extending incubation, and quantifying fluorescence on a microplate reader. Annexin V binding was used as a qualitative indicator of phosphatidylserine externalization during early apoptosis. Annexin V fluorescence was evaluated as a comparative semi-quantitative assessment under identical acquisition settings for all experimental groups. Therefore, these measurements should not be interpreted as definitive quantification od apoptotic cell death.

**Western Blot**

Caco-2 cells (8 × 10^5^ per well) were seeded in 6-well plates and treated for 48 h with BGE (15.63 and 31.25 μg/mL), cisplatin (37.5 μM), combinations with BGE or betanin, and purified betanin (12.5 μM). Cells were lysed using RIPA buffer (Thermo Fisher Scientific, Waltham, MA, USA), and protein concentration was determined using the Bradford method (Pierce Detergent Compatible Bradford Assay Kit, Thermo Scientific), with a bovine serum albumin (BSA) standard curve. Equal amounts of protein were separated by 10% SDS-PAGE electrophoresis for 1-2 h and transferred onto nitrocellulose membranes using transfer buffer. Membranes were blocked for 1 h at room temperature using 3% non-fat milk prepared in PBS-Tween 20 0.1%) before antibody incubation. The membranes were incubated overnight at 4°C with primary antibodies against Bcl-2 (1:2000), p53 (1:5000), and β-actin-HRP (1:10,000). After washing with PBS-Tween 20, membranes were incubated with HRP-conjugated anti-mouse secondary antibody (1:10,000) for 1 h at room temperature. Protein bands were visualized using TMB substrate (Thermo Fisher Scientific, Waltham, MA, USA). Relative protein expression was quantified by densitometric analysis using Fiji/ImageJ software after normalization to β-actin.

**Apoptosis Gene Expression Analysis**

Caco-2 cells were treated with BGE (31.25 μg/ml) and negative control for 48h at 37°C and lysed. RNA was extracted using the QIAwave RNA Mini Kit (Qiagen). cDNA synthesis was performed using the High-Capacity RNA-to-cDNA™ Kit (Applied Biosystems, Thermo Scientific). RT-PCR was performed according to the TaqMan™ Array Human Apoptosis kit protocol using QuantGene 9600 Fluorescent Quantitative Detection System (Hangzhou Bioer Technology Co). Relative gene expression was calculated using the comparative ΔΔCt method. Ct values for target genes were normalized against the endogenous housekeeping gene included in the TaqMan assay system (GUSB), and fold changes were calculated relative to untreated control cells.

**Statistical Analysis**

The results were analyzed using one-way analysis of variance (ANOVA), a two-group comparison with a t-test, and a Tukey comparison test on Minitab Statistical Software. The statistically significant value was set as p < 0.05. The given data represent the mean ± standard deviation of replicates. All tests were performed in triplicate, except for the TaqMan Array Human Apoptosis analysis. Statistical analyses of gene expression were performed using R (version 3.6.0).

**Supplementary Information (Tables)**

**Table S1. Chromatographic and mass spectrometric analytical conditions used for the identification and quantification of phenolic compounds in the *Bougainvillea glabra* extract (BGE) using authentic reference standards**

| **Compound** | **dMRM transition** | | | **Mass spectrometric conditions** | | | **Quantification conditions** | | |
| --- | --- | --- | --- | --- | --- | --- | --- | --- | --- |
|  | **Precursor ion** | **Product ion** | **Retention time** | **Collision energy** | **Fragmentor** | **Polarity** | **Quantification range (µM)** | **Regression type** | **R^2^** |
| Shikimic acid^1^ | 173.1 | 111.1 | 0.53 | 10 | 100 | Negative | 0.5 - 19 | Quadratic | 0.99 |
| Gallic acid^2^ | 169.0 | 125.2 | 1.56 | 10 | 100 | Negative | 0.5 - 19 | Quadratic | 0.99 |
| L-Phenylalanine^3^ | 166.1 | 131.0 | 2.16 | 10 | 100 | Positive | 0.5 - 19 | Quadratic | 0.99 |
| Protocatechuic acid^1^ | 153.0 | 109.1 | 2.91 | 10 | 100 | Negative | 0.5 - 19 | Quadratic | 0.99 |
| 4-Hydroxybenzoic acid^1^ | 137.1 | 92.8 | 4.38 | 10 | 100 | Negative | 0.5 - 19 | Quadratic | 0.99 |
| Gentisic acid^1^ | 153.0 | 109.0 | 4.44 | 10 | 100 | Negative | 0.5 - 19 | Quadratic | 0.99 |
| Vainilloside^2^ | 315.1 | 153.0 | 4.94 | 10 | 100 | Positive | 0.5 - 19 | Quadratic | 0.99 |
| 4-Hydroxyphenylacetic acid^3^ | 107.1 | 77.0 | 5.38 | 20 | 140 | Positive | 0.5 - 19 | Quadratic | 0.99 |
| (-)-Epigallocatechin^4^ | 305.1 | 125.0 | 5.44 | 20 | 140 | Negative | 0.5 - 19 | Quadratic | 0.99 |
| (+)-Catechin^2^ | 291.0 | 138.9 | 5.73 | 10 | 100 | Positive | 0.5 - 19 | Quadratic | 0.99 |
| Vanillic acid^1^ | 169.0 | 93.0 | 5.93 | 10 | 100 | Positive | 0.5 - 19 | Quadratic | 0.99 |
| Chlorogenic acid^1^ | 355.1 | 163.0 | 6.14 | 10 | 100 | Positive | 0.5 - 19 | Quadratic | 0.99 |
| Caffeic acid^1^ | 181.0 | 163.0 | 6.15 | 10 | 100 | Positive | 0.5 - 19 | Quadratic | 0.99 |
| Scopolin^5^ | 355.1 | 193.0 | 6.18 | 20 | 100 | Positive | 0.5 - 19 | Quadratic | 0.99 |
| Malvin^1^ | 655.1 | 331.1 | 6.42 | 40 | 100 | Positive | 0.5 - 19 | Quadratic | 0.99 |
| Kuromanin^1^ | 449.0 | 286.9 | 6.98 | 30 | 100 | Positive | 0.5 - 19 | Quadratic | 0.99 |
| Procyanidin B2^1^ | 577.1 | 425.1 | 7.24 | 10 | 100 | Negative | 0.5 - 19 | Quadratic | 0.99 |
| Keracyanin^2^ | 595.2 | 287.1 | 7.56 | 20 | 100 | Positive | 0.5 - 19 | Quadratic | 0.99 |
| Vanillin^1^ | 153.0 | 124.9 | 7.65 | 10 | 100 | Positive | 0.5 - 19 | Quadratic | 0.99 |
| (-)-Epicatechin^2^ | 291.0 | 138.8 | 7.68 | 10 | 100 | Positive | 0.5 - 19 | Quadratic | 0.99 |
| Mangiferin^2^ | 423.0 | 302.8 | 8.00 | 10 | 100 | Positive | 0.5 - 19 | Quadratic | 0.99 |
| 4-Coumaric acid^1^ | 165.0 | 147.0 | 8.23 | 10 | 100 | Positive | 0.5 - 19 | Quadratic | 0.99 |
| (-)-Gallocatechin gallate^2^ | 458.9 | 139.0 | 8.74 | 20 | 80 | Positive | 0.5 - 19 | Quadratic | 0.99 |
| Umbelliferone^1^ | 163.0 | 107.0 | 8.81 | 30 | 100 | Positive | 0.5 - 19 | Quadratic | 0.99 |
| Quercetin 3,4-di-O-glucoside^1^ | 627.0 | 302.9 | 9.51 | 10 | 100 | Positive | 0.5 - 19 | Quadratic | 0.99 |
| Scopoletin^1^ | 193.0 | 133.0 | 9.63 | 10 | 100 | Positive | 0.5 - 19 | Quadratic | 0.99 |
| Ferulic acid^1^ | 195.1 | 145.0 | 9.69 | 20 | 100 | Positive | 0.5 - 19 | Quadratic | 0.99 |
| 3-Coumaric acid^1^ | 165.05 | 147.04 | 9.78 | 10 | 100 | Positive | 0.5 - 19 | Quadratic | 0.99 |
| Sinapic acid^1^ | 225.1 | 207.1 | 10.26 | 10 | 100 | Positive | 0.5 - 19 | Quadratic | 0.99 |
| Salicylic acid^2^ | 137.0 | 93 | 10.70 | 10 | 100 | Negative | 0.5 - 19 | Quadratic | 0.99 |
| Epicatechin gallate^4^ | 443.1 | 123.0 | 10.71 | 10 | 100 | Positive | 0.5 - 19 | Quadratic | 0.99 |
| Ellagic acid^1^ | 300.5 | 145.0 | 10.72 | 30 | 170 | Negative | 0.5 - 19 | Quadratic | 0.99 |
| Myricitrin^1^ | 465.0 | 318.9 | 10.82 | 10 | 100 | Positive | 0.5 - 19 | Quadratic | 0.99 |
| Quercetin 3-D-galactoside^2^ | 465.0 | 302.9 | 11.09 | 10 | 100 | Positive | 0.5 - 19 | Quadratic | 0.99 |
| Rutin^2^ | 611.0 | 302.9 | 11.12 | 10 | 100 | Positive | 0.5 - 19 | Quadratic | 0.99 |
| Quercetin 3-glucoside^2^ | 465.0 | 303.0 | 11.38 | 10 | 100 | Positive | 0.5 - 19 | Quadratic | 0.99 |
| Luteolin 7-O-glucoside^1^ | 449.0 | 287.0 | 11.60 | 10 | 100 | Positive | 0.5 - 19 | Quadratic | 0.99 |
| *p*-Anisic acid^3^ | 153.1 | 109.0 | 11.68 | 5 | 120 | Positive | 0.5 - 19 | Quadratic | 0.99 |
| Penta-O-galloyl-B-D-glucose^2^ | 771.1 | 153.0 | 12.45 | 20 | 100 | Positive | 0.5 - 19 | Quadratic | 0.99 |
| 2,4-Dimethoxy-6-methylbenzoic acid^2^ | 197.0 | 179.0 | 12.57 | 5 | 80 | Positive | 0.5 - 19 | Quadratic | 0.99 |
| Kaemperol 3-O-glucoside^1^ | 449.0 | 286.9 | 12.76 | 10 | 100 | Positive | 0.5 - 19 | Quadratic | 0.99 |
| Quercitrin^1^ | 449.1 | 303.1 | 12.79 | 10 | 100 | Positive | 0.5 - 19 | Quadratic | 0.99 |
| Naringin^1^ | 273.0 | 153.0 | 12.91 | 10 | 120 | Positive | 0.5 - 19 | Quadratic | 0.99 |
| Myricetin^1^ | 317.0 | 179.0 | 13.16 | 10 | 100 | Negative | 0.5 - 19 | Quadratic | 0.99 |
| Hesperidin^1^ | 609.1 | 301.1 | 13.48 | 20 | 100 | Negative | 0.5 - 19 | Quadratic | 0.99 |
| Rosmarinic acid^1^ | 361.1 | 163.0 | 13.62 | 10 | 100 | Positive | 0.5 - 19 | Quadratic | 0.99 |
| *trans*-Resveratrol^2^ | 229.1 | 135.0 | 13.81 | 10 | 100 | Positive | 0.5 - 19 | Quadratic | 0.99 |
| Phloridzin^1^ | 435.0 | 272.9 | 13.84 | 10 | 100 | Negative | 0.5 - 19 | Quadratic | 0.99 |
| Secoisolariciresinol^2^ | 363.2 | 137.1 | 13.98 | 20 | 100 | Positive | 0.5 - 19 | Quadratic | 0.99 |
| *trans*-Cinnamic acid^1^ | 149.1 | 131.0 | 15.31 | 10 | 100 | Positive | 0.5 - 19 | Quadratic | 0.99 |
| Quercetin^2^ | 301 | 151 | 16.17 | 20 | 100 | Negative | 0.5 - 19 | Quadratic | 0.99 |
| Luteolin^1^ | 285 | 151 | 16.33 | 20 | 100 | Negative | 0.5 - 19 | Quadratic | 0.99 |
| Psoralen^1^ | 187.0 | 131.1 | 16.82 | 20 | 100 | Positive | 0.5 - 19 | Quadratic | 0.99 |
| Angelicin^2^ | 187.0 | 131.1 | 17.49 | 20 | 100 | Positive | 0.5 - 19 | Quadratic | 0.99 |
| Naringenin^1^ | 271.0 | 151 | 17.97 | 10 | 100 | Negative | 0.5 - 19 | Quadratic | 0.99 |
| Apigenin^1^ | 271.0 | 153.0 | 18.68 | 30 | 100 | Positive | 0.5 - 19 | Quadratic | 0.99 |
| Kaempferol^1^ | 285 | 151 | 18.99 | 10 | 100 | Negative | 0.5 - 19 | Quadratic | 0.99 |
| Hesperetin^1^ | 303.1 | 177.1 | 19.26 | 20 | 100 | Positive | 0.5 - 19 | Quadratic | 0.99 |
| Podophyllotoxin^4^ | 415.1 | 397.1 | 20.18 | 10 | 100 | Positive | 0.5 - 19 | Quadratic | 0.99 |
| Methyl cinnamate^2^ | 163.1 | 131.0 | 23.21 | 6 | 100 | Positive | 0.5 - 19 | Quadratic | 0.99 |
| Nordihydroguaiaretic acid^1^ | 303.0 | 193.1 | 23.73 | 10 | 100 | Positive | 0.5 - 19 | Quadratic | 0.99 |
| Chrysin^1^ | 255.1 | 153.0 | 24.37 | 40 | 100 | Positive | 0.5 - 19 | Quadratic | 0.99 |
| Kaempferide^1^ | 299 | 284 | 25.75 | 20 | 100 | Positive | 0.5 - 19 | Quadratic | 0.99 |
| Emodin^1^ | 269.0 | 225.0 | 28.79 | 20 | 150 | Negative | 0.5 - 19 | Quadratic | 0.99 |
| Chrysophanol^1^ | 255.1 | 153.0 | 32.47 | 40 | 100 | Positive | 0.5 - 19 | Quadratic | 0.99 |

**Table S2. Tentatively identified compounds detected in *Bougainvillea glabra* extract (BGE) by non-targeted LC-MS profiling.** Compound annotation was based on accurate mass measurements, fragmentation patterns, and comparison with literature reports and database entries.

| Retention time (min) | m/z (Da) | Name | Formula | Monoisotopic molecular weight (Da) | Error (ppm) |
| --- | --- | --- | --- | --- | --- |
| 1.30 | 268.1046 | Adenosine | C_10_H_13_N_5_O_4_ | 267.0968 | 2 |
| 1.36 | 294.1547 | N-(1-Deoxy-1-fructosyl)leucine | C_12_H_23_NO_7_ | 293.1475 | 0 |
| 1.83 | 328.1394 | N-(1-Deoxy-1-fructosyl)phenylalanine | C_15_H_21_NO_7_ | 327.1318 | 1 |
| 2.36 | 367.1495 | N-(1-Deoxy-1-fructosyl)tryptophan | C_17_H_22_N_2_O_7_ | 366.1427 | 1 |
| 2.44 | 205.0970 | Tryptophan | C_11_H_12_N_2_O_2_ | 204.0899 | 1 |
| 3.52 | 773.2137 | Quercetin 3-O-glucosyl-rutinoside | C_33_H_40_O_21_ | 772.2062 | 4 |
| 3.90 | 649.1370 | Quercetin 3,5-diglucoside | C_27_H_30_O_17_ | 626.1483 | 1 |
| 4.38 | 611.1604 | Rutin | C_27_H_30_O_16_ | 610.1534 | 0 |
| 4.45 | 663.1530 | Isorhamnetin 3,4'-diglucoside | C_28_H_32_O_17_ | 640.1639 | 0 |
| 4.53 | 641.1713 | Isorhamnetin 3,7-diglucoside | C_28_H_32_O_17_ | 640.1639 | 0 |
| 4.96 | 647.1575 | Isorhamnetin rutinoside | C_28_H_32_O_16_ | 624.1690 | 1 |
| 5.06 | 501.1003 | Isorhamnetin hexoside | C_22_H_22_O_12_ | 478.1111 | 0 |
| 5.15 | 317.0661 | Isorhamnetin | C_16_H_12_O_7_ | 316.0583 | 2 |
| 6.95 | 573.1325 | Betanin | C_24_H_26_N_2_O_13_ | 550.1435 | 0 |
| 7.36 | 411.0807 | Betanidin | C_18_H_16_N_2_O_8_ | 388.0907 | 1 |

**Table S3. Measured-to-expected ratios for the combined effects of Bougainvillea glabra extract (BGE) or betanin with cisplatin in Caco-2 cell viability**

|  | Combination |  | Composition (µg/mL BGE/µM Betanin) | Measured/Expected Ratio |
| --- | --- | --- | --- | --- |
| Extract | C1 |  | 125.0 | 1.00* |
|  | C2 |  | 62.5 | 1.33* |
|  | C3 |  | 31.2 | 1.26* |
|  | C4 |  | 15.6 | 0.85 |
| Purified betanin | C1 |  | 50.0 | 0.96 |
|  | C2 |  | 25.0 | 0.94 |
|  | C3 |  | 12.5 | 0.94 |

*Values marked with an asterisk (*) indicate statistically significant differences according to Student’s t-test (p < 0.05) between cisplatin alone and the corresponding combined treatment with BGE or betanin.

**Supplementary Information (Figures)**

**Fig. S1 Spectrophotometric screening of *Bougainvillea glabra* extract (BGE).** The absorption spectrum showed two major absorption peaks at approximately 280 nm and 530 nm, corresponding to phenolic compound-associated absorption and betacyanin pigments, respectively. Absorbance values near 280 nm exceeded the detection range of the instrument, resulting in partial truncation of the peak.

**Fig. S2 Representative LC-MS chromatogram obtained from non-targeted chemical profiling of *Bougainvillea glabra* extract (BGE).** Tentatively identified compounds are indicated by arrows and corresponding peak numbers. Compounds annotation was based on mass spectral data, fragmentation patterns, and comparison with literature reports and database entries.

**Fig. S3 Trolox calibration curve used for ABTS radical scavenging assay quantification.** Trolox solutions of known concentrations were used to generate the calibration curve employed for the calculation of Trolox equivalent (TE) values in the ABTS radical scavenging assay. The linear regression equation and coefficient of determination (R²) are shown.

**Fig. S4 Effects of BGE and cisplatin on HDFa cell viability for 48 h.** Cell viability was calculated relative to untreated control cells. Data are presented as mean ± SD (n = 3). Different letters indicate statistically significant differences according to Tukey’s post hoc test (p < 0.05).

**Fig. S5 Effects of BGE, betanin, cisplatin, and their combinations on autophagic-associated fluorescence in Caco-2 cells.** Relative fluorescence intensity associated with autophagic vesicle staining was evaluated using a fluorescent autophagosome probe and normalized to the number of Hoechst-positive nuclei per field using Fiji/ImageJ analysis. Results are expressed as fold change relative to untreated control cells. Rapamycin (500 nM) was used as a positive control, whereas cisplatin (18.75 µM) was used as a reference treatment to induce detectable autophagy-associated fluorescence without excessive cytotoxicity. Data are expressed as mean ± SD from representative fields and should be interpreted as semi-quantitative analysis.

**Fig. S6 Apoptosis-associated fluorescence in Caco-2 cells following treatment with BGE, betanin, cisplatin, and their combinations.** Apoptosis-associated fluorescence was evaluated using an Annexin V-based assay and expressed as fold change relative to untreated control cells. Cisplatin (37.5 µM) was included as a reference control. Data are presented as mean ± SD. Different letters indicate statistically significant differences according to Tukey’s post hoc test (p < 0.05).

**A**

**B** **C**

**Fig. S7 Immunoblot analysis of apoptosis-related proteins p53 and Bcl-2 in Caco-2 cells following treatment with BGE, betanin, cisplatin, and their combinations.** β-actin was used as a loading control. Molecular weights are indicated on the right side of the blot. Relative protein expression was quantified by densitometric analysis using Fiji/ImageJ software after normalization to β-actin. Panels **B** and **C** show the relatively normalized expression of p53 and Bcl-2. Data are shown as relative expression; no statistical analysis was performed due to a single independent experiment.

**References**

1.	Singleton VL, Orthofer R, Lamuela-Raventós RM (1999) Analysis of total phenols and other oxidation substrates and antioxidants by means of Folin-Ciocalteu reagent. pp 152–178

2.	Monribot-Villanueva JL, Altúzar-Molina A, Aluja M, et al (2022) Integrating proteomics and metabolomics approaches to elucidate the ripening process in white Psidium guajava. Food Chemistry 367:130656. https://doi.org/10.1016/j.foodchem.2021.130656

3.	Elisia I, Kitts DD (2008) Anthocyanins inhibit peroxyl radical-induced apoptosis in Caco-2 cells. Mol Cell Biochem 312:139–145. https://doi.org/10.1007/s11010-008-9729-1

4.	Farabegoli F, Scarpa ES, Frati A, et al (2017) Betalains increase vitexin-2-O-xyloside cytotoxicity in CaCo-2 cancer cells. Food Chemistry 218:356–364. https://doi.org/10.1016/j.foodchem.2016.09.112
